# The Faddist in Dentistry

**Published:** 1905-02

**Authors:** 


### Editorial.

### THE FADDIST IN DENTISTRY.

It is a peculiar trait in human nature to run in droves and to become infatuated with one thing until exhausted. This is common to the race, but more pronounced in some sections than in others. This may be due to climatic conditions, and possibly it may furnish the explanation why the people in this part of the world exhibit this trait to a greater extent than more conservative peoples elsewhere. "To run mad" after a new thing is a common expression, but it very nearly describes the mental attitude of the masses under the hypnotic influence of a peculiar stimulus.

This wild enthusiasm in the adoption of a new idea, which so much prevails, is not without its redeeming quality. Were it not for this, much that has made the new life in this country so full of vigor, with a readiness to receive new impressions and to evolve new conceptions, would have been lost to the world. Indeed, it is feared that modern civilization is hardly cognizant of the debt due to this vigorous concentration of mind in the investigation of unsolved problems, so prevailing in American life. The new inventions, the discoveries that have had their origin here, and which have so much influenced the civilization of the nineteenth century, could not have been accomplished but for this spirit that has become intense with life in this people and is beginning to infuse its power into the conservative elements of older civilizations.

That this has induced an enthusiastic tendency is not singular, for it may be found in all phases of mental activity. It only requires a leader of unusual mental force to point out the way, to be followed by a rush of converts, whether this be to the temples of religion, to the doubtful arena of politics, or the more quiet avenues of science and philosophy.

The history of dentistry is replete with this tendency to grow unduly enthusiastic over a new theory, new method, new process, or new appliance. The earlier history of pulp-capping is very instructive and full of minor instances of this tendency to hastily accept a new idea without due reflection. How many methods were devised for the salvation of pulps, and how the dental operators would go, almost in a body, to examine and adopt this new revelation, to be forced, in the end, to acknowledge that empiricism led where experience and wisdom demanded results.

Perhaps no better evidence of the faddist tendency was ever given in dentistry than was manifested upon the introduction of cohesive gold. Dentists became enthused with this as the greatest blessing ever vouchsafed to an overworked body of men. Everything could be accomplished with this, it was asserted, that had ever been done with non-cohesive gold, and that with less labor and with more certainty as to results, and we all, including the writer, became enthused over gold and nothing but gold. The huge fillings of that day, placed in with so much toil and skill, attest this almost insane devotion to one material. The conservative man who still adhered to old methods, with non-cohesive gold as the best for his patients and himself, was looked upon as non-progressive, and was the subject of the sympathy and pity of his associates.

This enthusiasm worked its way until more reasonable views began to change the minds of dental operators. They were forced to recognize that this supposed perfection in filling-material had its weak points. That it required more skill to insert and a sacrifice of time injurious to the operator and exhausting to patient, physically and financially, and this brought a return wave of mental objections resulting in the era of plastics. The much-abused amalgam, through the "New Departure" fad, became reinstated in favor, and men rushed into this old method of filling teeth, and the older ideas relating to gold for large cavities were regarded by many as an unwise procedure. No one claimed that amalgam was artistic, or even wholly satisfactory, but it relieved the operator and the patient of an onerous burden, and was, equally with gold, a preservative from decay. The amalgam craze has had its day, and is now on the decline; in fact, no one writes papers on its shrinkage, or how it may be avoided through certain alloys, or how the material should be inserted, yet its use will remain as one of the important materials for filling defective teeth.

It does not require a long memory to go back to the wave of enthusiasm and hope that filled the professional mind upon the introduction of the method of overcoming the hypersensitiveness of dentine through cataphoresis. The supply houses could not readily supply the demand for appliances, yet the unfortunate results are too fresh in the experience of many to need extended notice. It was unfortunate, for cataphoresis is, and must always remain, a valuable aid in the treatment of sensitive teeth, but it is not a process for ignorance to handle, and this was too much the case with the majority who attempted its use. The possible dangers of the electrical current in connection with a delicate organ, as the pulp, were not sufficiently understood, hence frequent devitalization followed, and cataphoresis was relegated to the few who appreciated its value and understood the proper methods of use.

Now we are in the storm centre of another method of filling. It has grown more slowly than any other fad in dentistry. It began years ago at a time when dentists would aim to fit a portion of porcelain into cavities on the labial surfaces of incisors, and for years this was the ultimate of the ambitious practitioner. Suddenly a tidal wave, figuratively, came across the Atlantic, and, singularly, not from a foreign mind, but from the progressive intellect of an American, weary with the inartistic productions of gold. The Jenkins low-fusing body started a furor for all sorts of inlays, low fusing, high fusing, new combinations of porcelain, new methods of forming matrices, shaping cavities, etc., and very little is heard now in our conventions but porcelain for all sorts and sizes of cavities. So much has this developed that the average dentist cares but little for anything but this and bridge-work. These are the fads of the hour.

The programme of a prominent dental society lies before the writer, in which the meeting, it is stated, will begin with a paper on "Porcelain Problems," to be followed by fifteen separate clinics on porcelain inlays and crowns, with two of gold inlays. Thus an entire afternoon and evening will be consumed with this subject. No objection could be made to this if it did not sacrifice, for the time, all interest in the scientific side of dentistry, so important in its several relations.

It must be said, however, that this porcelain craze should be encouraged, for, in proportion as it becomes perfected, will be removed from dentistry one of its most laborious operations, as well as the opprobrium heaped upon it for its disfigurement of the mouth through a lack of artistic methods. If it can infuse a better taste in our operative work, it will be worth all the time spent in its development.

The enthusiasm of the faddist is not, therefore, to be cast aside as unworthy a body of intelligent workers. It is the stimulus to progress, and should be encouraged in spite of possible injury produced through the crude efforts of the unskilled. There is a great stimulus in the combined mental effort of many. It forces the masses to active competition, and, while it may result in a relapse, it will leave an impress for good not to be eradicated by time, but will leave a healthier state than was manifest at the close of the nineteenth century.

That which is true of porcelain can be said equally of all the apparently wild impulses of dental workers. The results have justified the energy displayed, faulty though it may have been, and which has led the profession of dentistry upward to a higher standard of thought and practice.

